# Inhibition Studies with 2-Bromoethanesulfonate Reveal a Novel Syntrophic Relationship in Anaerobic Oleate Degradation

**DOI:** 10.1128/AEM.01733-18

**Published:** 2019-01-09

**Authors:** A. F. Salvador, A. J. Cavaleiro, A. M. S. Paulo, S. A. Silva, A. P. Guedes, M. A. Pereira, A. J. M. Stams, D. Z. Sousa, M. M. Alves

**Affiliations:** a Centre of Biological Engineering, University of Minho, Braga, Portugal; b Laboratory of Microbiology, Wageningen University and Research, Wageningen, Netherlands; Goethe University Frankfurt am Main

**Keywords:** 2-bromoethanesulfonate (BrES), *Desulfovibrio*, *Syntrophomonas*, desulfonation, isethionate, oleate, syntrophy

## Abstract

Degradation of long-chain fatty acids (LCFAs) in methanogenic environments is a syntrophic process involving the activity of LCFA-degrading bacteria and hydrogen-utilizing methanogens. If methanogens are inhibited, other hydrogen scavengers are needed to achieve complete LCFA degradation. In this work, we developed two different oleate (C_18:1_ LCFA)-degrading anaerobic enrichment cultures, one methanogenic (ME) and another in which methanogenesis was inhibited (IE). Inhibition of methanogens was attained by adding a solution of 2-bromoethanesulfonate (BrES), which turned out to consist of a mixture of BrES and isethionate. Approximately 5 times faster oleate degradation was accomplished by the IE culture compared with the ME culture. A bacterium closely related to Syntrophomonas zehnderi (99% 16S rRNA gene identity) was the main oleate degrader in both enrichments, in syntrophic relationship with hydrogenotrophic methanogens from the genera Methanobacterium and Methanoculleus (in ME culture) or with a bacterium closely related to Desulfovibrio aminophilus (in IE culture). A Desulfovibrio species was isolated, and its ability to utilize hydrogen was confirmed. This bacterium converted isethionate to acetate and sulfide, with or without hydrogen as electron donor. This bacterium also utilized BrES but only after 3 months of incubation. Our study shows that syntrophic oleate degradation can be coupled to desulfonation.

**IMPORTANCE** In anaerobic treatment of complex wastewater containing fat, oils, and grease, high long-chain fatty acid (LCFA) concentrations may inhibit microbial communities, particularly those of methanogens. Here, we investigated if anaerobic degradation of LCFAs can proceed when methanogens are inhibited and in the absence of typical external electron acceptors, such as nitrate, iron, or sulfate. Inhibition studies were performed with the methanogenic inhibitor 2-bromoethanesulfonate (BrES). We noticed that, after autoclaving, BrES underwent partial hydrolysis and turned out to be a mixture of two sulfonates (BrES and isethionate). We found out that LCFA conversion proceeded faster in the assays where methanogenesis was inhibited, and that it was dependent on the utilization of isethionate. In this study, we report LCFA degradation coupled to desulfonation. Our results also showed that BrES can be utilized by anaerobic bacteria.

## Abstract (precis)

In anaerobic treatment of complex wastewater containing fat, oils, and grease, high long-chain fatty acid (LCFA) concentrations may inhibit microbial communities, particularly those of methanogens. Here, we investigated if anaerobic degradation of LCFAs can proceed when methanogens are inhibited and in the absence of typical external electron acceptors, such as nitrate, iron, or sulfate. Inhibition studies were performed with the methanogenic inhibitor 2-bromoethanesulfonate (BrES). We noticed that, after autoclaving, BrES underwent partial hydrolysis and turned out to be a mixture of two sulfonates (BrES and isethionate). We found out that LCFA conversion proceeded faster in the assays where methanogenesis was inhibited, and that it was dependent on the utilization of isethionate. In this study, we report LCFA degradation coupled to desulfonation. Our results also showed that BrES can be utilized by anaerobic bacteria.

## INTRODUCTION

Long-chain fatty acids (LCFAs) are found in several types of waste and wastewater, and they can be converted to methane in anaerobic bioreactors (1, 2). Conversion of LCFAs by anaerobic sludge relies on syntrophic relationships between LCFA-degrading bacteria and hydrogenotrophic methanogens. However, when LCFA concentrations are high, methanogenic activity can be inhibited (3–7). Due to thermodynamic constraints, LCFA degradation does not proceed if the hydrogen released from β-oxidation is not consumed by methanogens (8, 9). However, methanogens are not the only possible syntrophic partners. For instance, if sulfate is present, sulfate-reducing bacteria (e.g., Desulfovibrio, Desulfomicrobium, and Desulforhabdus species) are able to outcompete methanogens for hydrogen (10).

The diverse microbial communities degrading LCFA in bioreactors have been explored in a number of studies, but few microorganisms can be directly linked to LCFA degradation (5, 11–13). Syntrophomonas species, although usually found in low abundance in these ecosystems, have been identified as key players in the conversion of LCFA, in syntrophy with hydrogenotrophic methanogens or sulfate-reducing bacteria (10, 11, 14).

Conversion of LCFAs was reported in continuous bioreactors where methanogenesis was inhibited by 2-bromoethanesulfonate (BrES) and in the absence of external inorganic electron acceptors (15). In that work, unsaturated LCFAs (e.g., oleate, C_18:1_) were converted to the corresponding saturated LCFA with two fewer carbon atoms (e.g., palmitate, C_16:0_), which was not further degraded (15). Motivated by these observations, we started batch incubations with and without BrES and verified that LCFA could be completely degraded (unpublished data).

BrES is the best-known methanogenic inhibitor and is used in several applied and fundamental studies in which methanogenic inhibition is required (16–20). BrES is a structural analog of methyl-coenzyme M, competing with this molecule in the methanogenic pathway and thus hindering methane formation (21, 22). Besides inhibition of methanogens, BrES also affects other microbes by changing microbial community structure or by stimulating acetate metabolism and homoacetogenesis (23–27). Besides, the reduction of the sulfonate moiety of BrES to sulfide by spore-forming sulfate-reducing bacteria was previously reported during anaerobic dechlorination of polychlorobiphenyls (28).

To get further insight on LCFA degradation when methanogenesis is inhibited, we developed long-term oleate-degrading microbial enrichments with and without BrES, which later turned out to be a mixture of BrES and isethionate. We hypothesize that by inhibition of methanogens, oleate degradation can be coupled to homoacetogenesis (forming acetate from hydrogen and carbon dioxide) or to sulfonate reduction, since some sulfate-reducing bacteria are able to utilize sulfonates as electron donors and/or electron acceptors (29–31). Different syntrophic interactions were established during oleate conversion in enrichments where methanogenesis was active or inhibited. The hydrogen-consuming partner enriched in the absence of methanogens was further isolated and characterized for its ability to utilize sulfonates.

## RESULTS

### Characterization of oleate-degrading enrichment cultures.

**FIG 1:**
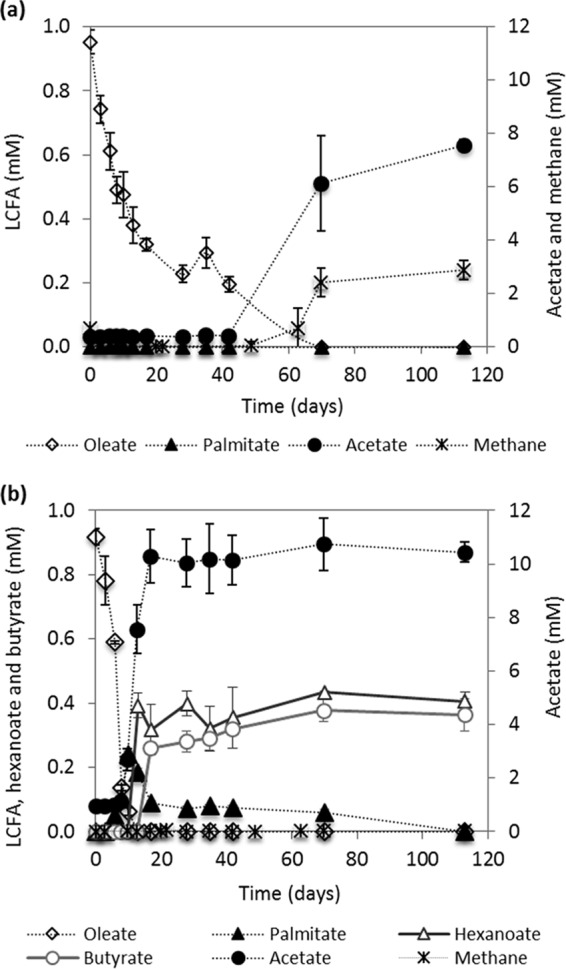
Oleate conversion by the enrichment cultures developed in the presence (a) and absence (b) of methanogenic activity. The results presented are the averages and standard deviations for triplicate assays.

Oleate-degrading enrichment cultures, one methanogenic (ME) and another in which methanogenesis was inhibited (IE), were obtained after five successive transfers. The two enrichments exhibited different oleate degradation rates and product profiles. The concentrations of oleate and the accumulation of the degradation products show that oleate conversion was faster in IE culture than in ME culture. Complete conversion of oleate in ME culture was achieved in approximately 113 days, while in IE culture, the same amount of oleate was degraded in 17 days (Fig. 1).

**FIG 2:**
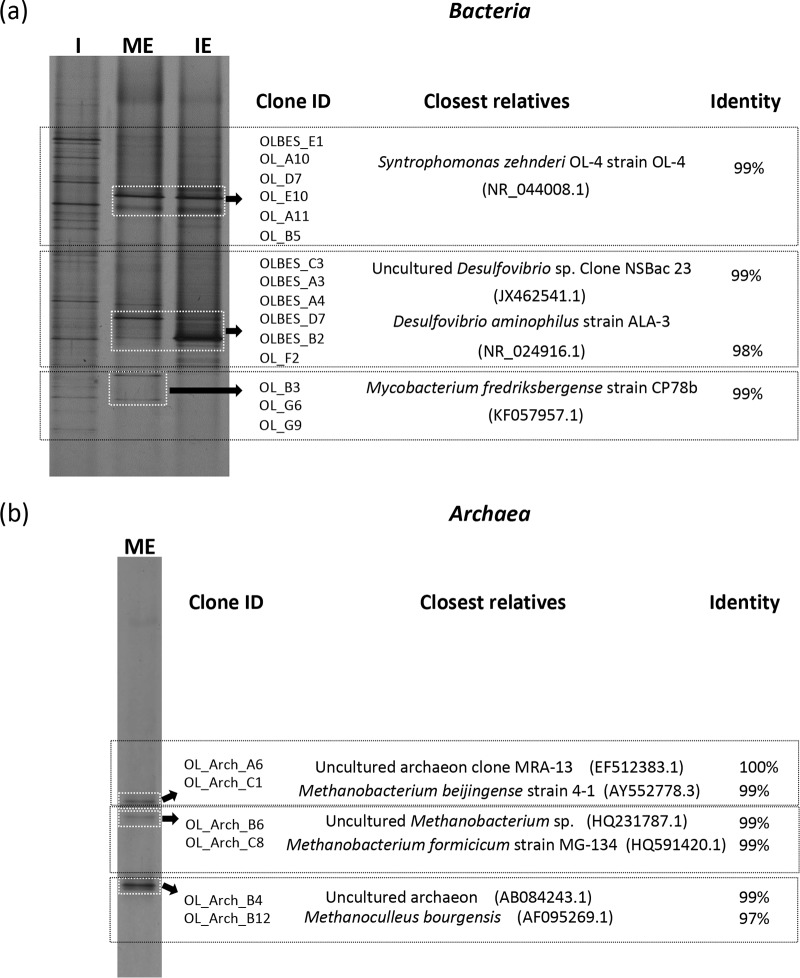
Taxonomic characterization of the microorganisms detected in oleate-degrading enrichments, given by DGGE fingerprinting and cloning and sequencing of bacterial (a) and archaeal (b) 16S rRNA genes. White squares delimit the DGGE bands corresponding to the 16S rRNA genes that were sequenced and further identified. The identity of the closest relatives and their corresponding sequence identifier is given (when the 16S rRNA gene sequence sharing the highest identity to the clone sequences is from an uncultivable microorganism, the identity of both microorganisms, the uncultivable and the first cultivable microorganism, is represented). I, inoculum sludge; ME, methanogenic enrichment; IE, enrichment culture in which methanogenesis was inhibited.

Under methanogenic conditions, oleate was converted to acetate (7.6 ± 0.2 mM) and approximately 3 mM methane (Fig. 1a), which corresponds to the maximum theoretical methane production, considering only hydrogenotrophic methanogenesis (Table 1, reaction 9). These results suggested that acetoclastic methanogens were absent or inhibited, which was confirmed by the identification of methanogens closely related to Methanobacterium beijingense, Methanobacterium formicicum, and Methanoculleus bourgensis, which are hydrogenotrophic. Typical acetoclastic methanogens (i.e., Methanosaeta and Methanosarcina species) were not detected by cloning and sequencing analysis (Fig. 2b).

**TABLE 1:** Possible reactions occurring during oleate conversion in the presence and absence of methanogenesis and during utilization of isethionate by *Desulfovibrio* sp.

Reaction no.	Equation and reaction	Δ*G*^0′^ (kJ/reaction)^*a*^
1	β-Oxidation of oleate	
	C_18_H_33_O_2_^−^ + 16H_2_O → 9C_2_H_3_O_2_^−^ + 15H_2_ + 8H^+^	325.86
2	Methanogenesis from hydrogen	
	4H_2_ + HCO_3_^−^ + H^+^ → CH_4_ + 3H_2_O	−135.58
3	Desulfonation of isethionate	
	C_2_H_5_O_4_S^−^ + H_2_O → C_2_H_3_O_2_^−^ + SO_3_^2−^ + 2H^+^ + H_2_	−48.70
4	Disproportionation of sulfite	
	4SO_3_^2−^ + H^+^ → 3SO_4_^2−^ + HS^−^	−235.52
4.1	Sulfite oxidation	
	SO_3_^2−^ + H_2_O → SO_4_^2−^ + H_2_	−20.83
4.2	Sulfite reduction	
	SO_3_^2−^ + 3H_2_ + H^+^ → HS^−^ + 3H_2_O	−173.03
5	Sulfate reduction	
	SO_4_^2−^ + 4H_2_ +H^+^ → HS^−^ + 4H_2_O	−152.20
6	Overall reaction for isethionate conversion without hydrogen	
	2C_2_H_5_O_4_S^−^ → 2C_2_H_3_O_2_^−^ + SO_4_^2−^ + HS^−^ + 3H^+^	−291.26
7	Overall reaction for isethionate conversion with hydrogen	
	C_2_H_5_O_4_S^−^ + 2H_2_ → C_2_H_3_O_2_^−^ + HS^−^ + H^+^ + 2H_2_O	−221.73
8	Overall reaction for oleate and isethionate conversion	
	C_18_H_33_O_2_^−^ + 7.5C_2_H_5_O_4_S^−^ + H_2_O → 16.5C_2_H_3_O_2_^−^ + 7.5HS^−^ + 15.5H^+^	−1,337.12
9	Overall reaction for methanogenic oleate conversion	
	C_18_H_33_O_2_^−^ + 4.75H_2_O + 3.75HCO_3_^−^ → 9C_2_H_3_O_2_^−^ + 3.75CH_4_ + 4.25H^+^	−182.57

a Δ*G*^0'^ was calculated under standard conditions (solute concentrations of 1 mol/liter, gas partial pressure of 1 × 10^5^ Pa, *T = *25°C) at pH 7. Free energies of formation for isethionate and oleate were estimated according to reference 51; for the other compounds involved in the reactions, the values were obtained from reference 52.

The IE culture produced acetate (10.4 ± 0.4 mM) and residual amounts of hexanoate and butyrate (<1 mM) from oleate degradation (Fig. 1b). Palmitate was detected at the beginning of the incubations, reaching a maximum of 0.2 mM at day 10, and was degraded afterwards (Fig. 1b). No methane was detected during oleate degradation.

Denaturing gradient gel electrophoresis (DGGE) analysis showed that bacterial diversity decreased greatly from the inoculum sludge to the final enrichment cultures (Fig. 2). Syntrophomonas (sharing 99% identity to the 16S rRNA gene of Syntrophomonas zehnderi strain OL-4) and Desulfovibrio (98% identity to Desulfovibrio aminophilus strain ALA-3) were present in both enrichments (Fig. 2a).

Thermodynamically, complete syntrophic oleate degradation can only occur if the electrons released by oleate-degrading bacteria are captured by another organism. In the ME culture, methanogens consumed the hydrogen, but in IE culture, the final electron acceptor was not known. To investigate the possibility that BrES could serve as electron acceptor, or that homoacetogenesis took place in IE culture, attempts were made to isolate hydrogen-utilizing microorganisms in anaerobic medium containing BrES.

The composition of the BrES solution was analyzed, and we verified that, after autoclaving, part of this compound was hydrolyzed to isethionate. Isethionate (HO-CH_2_-CH_2_-SO_3_^−^) is a sulfonate with the same structure as BrES (Br-CH_2_-CH_2_-SO_3_^−^), but with the bromide ion replaced by a hydroxide ion. After one cycle of sterilization by autoclavation, 16% ± 1.5% of the bromide is released from BrES, meaning that circa 16% of BrES was hydrolyzed to isethionate (Table S1). Both sulfonates could serve as electron acceptors for the conversion of oleate with sulfide production, and the sulfate-reducing bacteria identified in the IE culture (i.e., Desulfovibrio sp.) (Fig. 2) could probably be involved in sulfonate reduction.

### Isolation and characterization of the hydrogen scavenger enriched in the absence of methanogens.

The bacterium belonging to the genus Desulfovibrio was isolated after several transfers and serial dilutions of the IE culture, performed in anaerobic medium containing H_2_/CO_2_ and autoclaved BrES. To get a more reliable taxonomic identification of the isolate (here designated Desulf-BrES), the nearly complete 16S rRNA gene (approximately 1,400 bp) was sequenced and aligned with 16S rRNA sequences from the NCBI nucleotide collection database and from the Ribosomal Database Project (RDP) database. From the 90 clones obtained, 13 different clone sequences were retrieved, which shared a minimum of 99.12% and a maximum of 99.93% identity with each other (Fig. S2). Desulfovibrio aminophilus strain ALA-3 shared the highest identity (98.58% ± 0.14%) of the 16S rRNA gene with Desulf-BrES (Fig. S2). Desulf-BrES probably represents a new Desulfovibrio species, since the percentage of identity is lower than 98.7%, which is the threshold to classify microorganisms as the same species (32). Also, a maximum of 4 gap opens and 22 mismatches were obtained when comparing the 16S rRNA gene of D. aminophilus and Desulf-BrES (Fig. S2).

**FIG 3:**
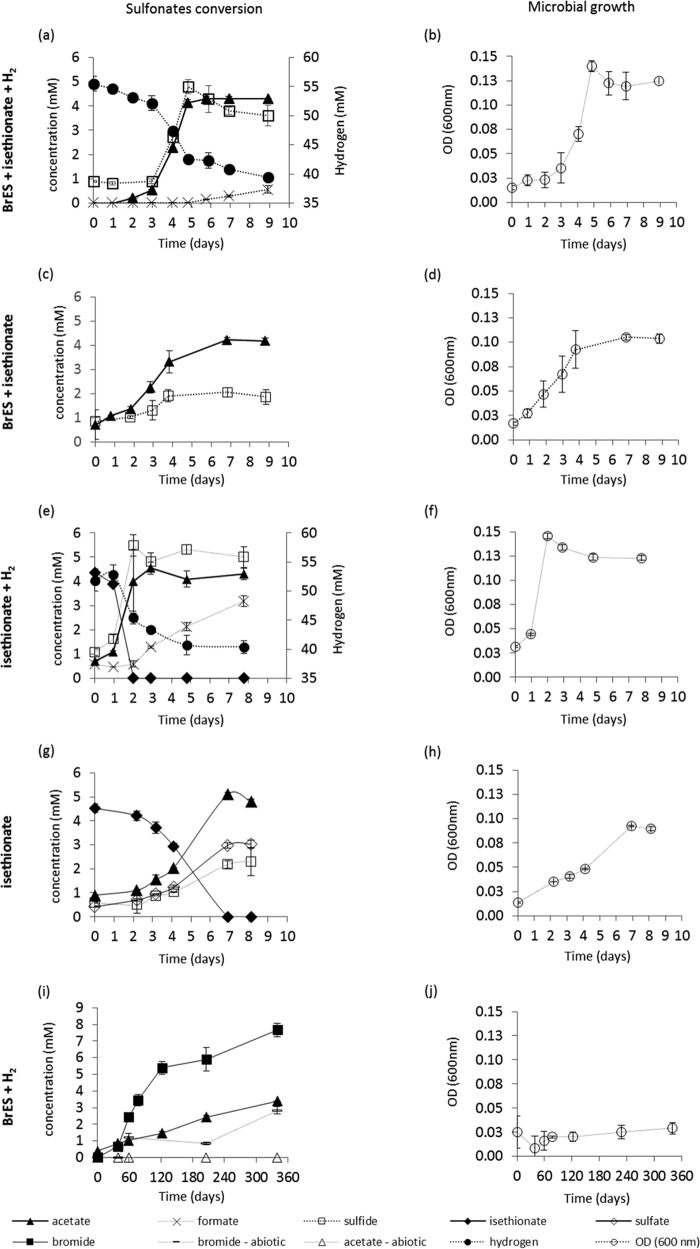
Sulfonate conversion by culture Desulf-BrES when incubated with (a) BrES plus isethionate and hydrogen, (c) BrES plus isethionate, (e) isethionate and hydrogen, (g) isethionate, and (i) BrES and hydrogen, showing the accumulation of free bromide and acetate in biotic and abiotic assays. Microbial growth determined through OD measurements at 600 nm for all of these conditions is also shown (panels b, d, f, h, and j, respectively). The results presented are the averages and standard deviations for triplicate assays.

After approximately 5 days of incubation in H_2_/CO_2_ and autoclaved BrES, the culture optical density increased to 0.140 ± 0.005, and 4.1 ± 0.1 mM acetate and 4.8 ± 0.3 mM sulfide were produced, together with the consumption of 13 ± 0.5 mM hydrogen (Fig. 3a and b). Extended incubation time led to the accumulation of small amounts of formate (less than 1 mM), which was not associated with cell growth (Fig. 3a and b).

The formation of sulfide indicates that the sulfonates present in the culture media (either BrES, isethionate or both) were utilized as electron acceptors by Desulfovibrio species. Further incubations carried out without hydrogen showed that culture Desulf-BrES could utilize sulfonates not only as electron acceptors but also as electron donors, producing sulfide and acetate (Fig. 3c). However, the rate of sulfide production is slightly lower in the absence of hydrogen (Fig. 3c). Additional assays performed in phosphate-buffered anaerobic medium (without bicarbonate or carbon dioxide addition) and without hydrogen, confirmed the degradation of sulfonates with formation of the same degradation products, i.e., acetate and sulfide (Fig. S3). However, repeated transfers in phosphate-buffered medium were accompanied by a reduction in the culture activity, because carbon dioxide is needed for cell matter synthesis (data not shown).

Control assays with hydrogen and carbon dioxide, but without sulfonates, did not result in growth or acetate production, indicating that culture Desulf-BrES has no homoacetogenic activity. The ability of culture Desulf-BrES to degrade oleate was tested in both the presence and absence of sulfonates, and no degradation occurred, even after prolonged incubation (data not shown).

Incubation of culture Desulf-BrES with increasing concentrations of sulfonates (BrES:isethionate, 84:16%) showed that the amount of acetate formed was directly proportional to sulfonate concentration, and that this activity was linked to microbial growth (Fig. S4). However, only approximately 20% of the sulfonate mixture was converted to acetate and sulfide. Additional incubations with isethionate (98%) as the only carbon and energy source revealed that this compound was completely utilized by culture Desulf-BrES as electron donor and as electron acceptor (Fig. 3g). The products from complete isethionate degradation were acetate, sulfate, and sulfide. In the incubations with isethionate and hydrogen, only acetate and sulfide were detected (Fig. 3e).

In incubations with BrES sterilized by filtering (which causes no transformation of BrES to isethionate) and hydrogen (as additional electron donor), acetate was produced (up to 3.4 mM), and free bromide ion accumulated (up to 7.7 mM) in the medium (Fig. 3i). However, utilization of BrES could only be detected after approximately 3 months of incubation, and it was not associated with detectable growth, since the optical density of the culture did not change (Fig. 3j) and only few cells could be observed by phase-contrast microscopy (data not shown). Free bromide ions were also detected in the abiotic assays, reaching a maximum of 3 mM after 340 days of incubation (Fig. 3i), which corresponds to the release of circa 15% of the bromide from the added BrES. Under those conditions, no acetate or other volatile fatty acids (VFAs) were formed.

## DISCUSSION

In this work, incubation of anaerobic sludge with oleate in the presence or absence of sulfonates (BrES plus isethionate) resulted in the enrichment of Syntrophomonas zehnderi, which is an obligate syntrophic bacterium that degrades LCFAs (33). In the ME culture, S. zehnderi converted oleate to acetate and hydrogen, and hydrogen was utilized by the hydrogenotrophs M. formicicum, M. beijingense, and M. bourgensis to produce methane (Fig. 1 and 2 and Table 1, reaction 9). In the IE culture, S. zehnderi and Desulfovibrio species were the only microorganisms detected. This suggests that oleate conversion was carried out by the syntrophic interaction between these two microorganisms, with formation of acetate as the main metabolic product (Fig. 1 and 2 and Table 1, reaction 8). Desulfovibrio sp. was most likely the hydrogen scavenger, allowing fast oleate degradation (Fig. 1b). Indeed, the conversion of oleate with isethionate is thermodynamically much more favorable (Δ*G*^0ʹ^= −1,337.12 kJ/reaction) than the conversion of oleate coupled to hydrogenotrophic methanogenesis (Δ*G*^0ʹ^= −182.57 kJ/reaction) (Table 1, reactions 8 and 9).

The S. zehnderi and Desulfovibrio sp. relationship was dependent on the simultaneous utilization of isethionate (which was present in the BrES solution) as electron acceptor by the Desulfovibrio sp. (Fig. 3, Table 1, reaction 8). Further investigation of the metabolism of sulfonates by the Desulfovibrio sp. (culture Desulf-BrES) showed that hydrogen utilization only occurred concomitantly with isethionate consumption. Therefore, oleate and isethionate (the carbon moiety) served as electron donors for Syntrophomonas and Desulfovibrio species, respectively, and isethionate (the sulfonate moiety) was the final electron acceptor (Fig. S6).

Incubation of the isolated Desulfovibrio sp. with isethionate as the sole carbon and energy source showed that it was converted to acetate, sulfate, and sulfide (Fig. 3g, Table 1, reaction 6). Most likely, sulfite (generated from isethionate desulfonation) (Table 1, reaction 3) undergoes disproportionation generating sulfate plus sulfide (Table 1, reactions 4, 4.1, and 4.2), which justifies the detection of stoichiometric amounts of sulfate and sulfide at the end of the incubations (Fig. 3g, reaction 4). In the incubations containing hydrogen and isethionate, the same products were detected, with exception of sulfate, showing that isethionate was completely reduced to sulfide (Fig. 3e, reaction 7). Therefore, the occurrence of these two reactions (sulfite disproportionation or sulfite reduction) is directly linked with the availability of hydrogen.

The finding that anaerobic LCFA degradation can be coupled to the reduction of sulfonates to sulfide is remarkable. A previous study with oleate-degrading methanogenic communities inhibited by addition of BrES revealed that oleate could be converted to palmitate, uncoupled from methanogenesis (15), but that the palmitate formed was not further degraded. The main difference between that study and our enrichment is that the BrES added to the bioreactors was not sterilized by autoclaving and so no isethionate was available to be used as electron acceptor.

Because LCFAs may inhibit methanogens in anaerobic bioreactors (3–7), the presence of other electron acceptors, such as sulfonates, may be an alternative way of ensuring LCFA degradation when methanogens are less active. Indeed, isethionates are ubiquitous in nature as they appear naturally in the squid axon, mammalian tissue, human urine, red algae, and orb spiders’ webs (30, 34). Sulfonates are also found in the formulation of pharmaceuticals, shampoos, and soaps (34), and some commercial soaps contain both sulfonates and LCFA in their composition (e.g., sodium isethionate together with an LCFA, such as sodium palmitate and sodium stearate). Therefore, isethionate may occur in wastewater treatment systems, and LCFA degradation coupled to isethionate reduction can possibly happen.

The ability of some sulfate-reducing bacteria to utilize sulfonates either as electron donors, electron acceptors, or both simultaneously has been described (Table 2) (29–31, 35, 36). A total of 12 microbial strains, affiliated with the genera Alcaligenes, Bilophila, Desulfobacterium, Desulfomicrobium, Desulfonispora, Desulfitobacterium, and Desulfovibrio, have been associated with sulfonate utilization. The majority utilize sulfonate as an electron acceptor, while only two species, Desulfovibrio sp. strain GRZCYSA and Desulfonispora thiosulfatigenes strain GKNTAU, simultaneously utilize sulfonates as electron donors and electron acceptors. Desulfovibrio sp. strain GRZCYSA is the most versatile and grows with several sulfonates (isethionate, cysteate, and aminomethanesulfonate), while D. thiosulfatigenes strain GKNTAU was reported to grow only in taurine (Table 2). Therefore, the Desulfovibrio strain isolated in this study is the second microorganism described to utilize isethionate as electron donor and acceptor. Assays with BrES (and lactate as electron donor) were performed with Desulfovibrio desulfuricans strain IC1, but no BrES utilization was reported (30).

**TABLE 2:** Utilization of sulfonates as electron donors and/or acceptors by anaerobic bacteria

Microorganism	Electron donor(s)	Electron acceptor(s)	Reference(s)
Desulfovibrio desulfuricans IC1	Lactate	Isethionate	30, 31, 35
	Lactate	Cysteate	30, 31
	Lactate	Sulfoacetaldehyde	30
	Formate	Isethionate	35
Desulfovibrio desulfuricans ATCC 29577	Lactate	Isethionate	31
Desulfovibrio sp. strain RZACYSA	Lactate	Isethionate	29
	Lactate	Cysteate	
	Lactate	Aminomethanesulfonate	
	Lactate	Taurine	
Desulfovibrio sp. strain GRZCYSA	Isethionate	Isethionate	
	Cysteate	Cysteate	
	Aminomethanesulfonate	Aminomethanesulfonate	
Alcaligenes sp. NKNTAU	Taurine	Nitrate	
Bilophila wadsworthia RZATAU	Formate	Taurine	
	Formate	Isethionate	
	Formate	Cysteate	
Desulfobacterium autotrophicans	Lactate	Cysteate	30
Desulfomicrobium baculatum	Lactate	Isethionate	30
	Lactate	Cysteate	
Desulfonispora thiosulfatigenes strain GKNTAU	Taurine	Taurine	36
Desulfitobacterium sp. strain PCE 1	Lactate	Isethionate	35
	Lactate	Cysteate	
Desulfitobacterium dehalogenans	Lactate	Isethionate	35
	Lactate	Cysteate	
Desulfitobacterium hafniense	Lactate or pyruvate	Isethionate	31, 35

Microbial degradation of BrES was found under aerobic conditions in microbial fuel cells (37), but it could not convincingly be shown under anaerobic conditions. Sulfide production in microbial enrichments containing BrES was previously reported, but there is no information as to whether BrES was autoclaved or not (38). In another study, sulfide accumulated in pasteurized microbial cultures incubated with BrES, suggesting that it can be used as an electron acceptor (28). However, in the latter study, BrES was autoclaved, and, taking into consideration that part of BrES is converted to isethionate after autoclaving (as found in this study), it cannot be ruled out that isethionate and not BrES was reduced by those microbial communities.

The Desulfovibrio sp. isolated in this study was able to convert BrES to acetate, but only after very long incubation times (approximately 17% of BrES was converted to acetate after approximately 11 months of incubation [Fig. 3i]). Compared to isethionate, BrES was far more difficult to degrade. The results show that BrES, apart from a methanogenic inhibitor, can also be a metabolic target for sulfonate-utilizing microorganisms. However, the main changes in metabolic pathways caused by BrES consumption are likely to occur only in long-term incubations, due to the recalcitrant nature of BrES. Nevertheless, care should be taken when interpreting experiments performed with the methanogenic inhibitor BrES, especially when it is sterilized by autoclaving, since it can be converted to isethionate, which is a good substrate for bacteria, such as sulfate-reducing bacteria capable to metabolize sulfonates.

The enrichment strategy allowed us to uncover the role of specific microorganisms within microbial communities degrading oleate. Under anaerobic conditions, oleate was converted to methane by syntrophic communities of acetogenic bacteria and methanogens, but when methanogenesis was inhibited, another syntrophic relationship took place. In the presence of isethionate, oleate could be completely converted to acetate by the synergistic activity of fatty acid-degrading bacteria and sulfate-reducing bacteria capable of sulfonate metabolism. Since there are several microorganisms that use sulfonates as electron acceptors, syntrophic LCFA degradation coupled to sulfonate reduction may be widespread.

## MATERIALS AND METHODS

### Enrichment of oleate-degrading microbial cultures.

Suspended anaerobic sludge (ETAR do Freixo, Porto, Portugal) was acclimatized to LCFA in a mesophilic (37°C) anaerobic bioreactor (2.8 liters) working in continuous mode during 15 days. The feeding was a mixture of LCFA (1 mM total concentration) composed of 41% oleate, 44% stearate, 14% palmitate, and 1% myristate, supplemented with macronutrients, micronutrients, and sodium bicarbonate, as described elsewhere (39). An organic loading rate of 1 g chemical oxygen demand (COD) · liter^−1^ · day^−1^ and a hydraulic retention time of 1 day were applied. Once acclimated, the biomass was washed with anaerobic medium and incubated in batch at 37°C over 25 days, until all of the accumulated substrate was consumed. This sludge was then used as inoculum for the development of two distinct enrichments, one methanogenic (ME) and another in which methanogenesis was inhibited (IE) (Fig. S5). Inhibition of methanogenesis was achieved by adding 2-bromoethanesulfonate (BrES) (98%; Sigma-Aldrich, St. Louis, MO) at a final concentration of 20 mM. BrES was sterilized by autoclaving (1 bar, 121°C, 20 min). Incubations were done aseptically and under strict anaerobic conditions, as described elsewhere (6). The anaerobic medium was bicarbonate buffered (18), the headspace of the vials was pressurized with N_2_/CO_2_ (80%:20%, vol/vol) at 1.7 × 10^5^ Pa, and the medium was sterilized by autoclaving (1 × 10^5^ Pa, 121°C, 20 min). Before inoculation, the medium was supplemented with salts and vitamins as described by Stams et al. (18) and reduced by addition of sodium sulfide (0.8 mM), and sodium oleate (99%; Sigma-Aldrich, St. Louis, MO) was supplemented at a final concentration of 1 mM. All stock solutions were flushed with N_2_ prior to autoclaving.

### Characterization of oleate-degrading enrichment cultures.

Stable enrichments, ME and IE cultures, were obtained after 5 successive transfers (10%, vol/vol), over a period of 1 year. LCFA, VFA, and methane were monitored during the time course of oleate degradation. Total RNA was isolated for taxonomic characterization of the active fraction of the microbial communities. The microbial communities’ dynamics were followed by 16S rRNA gene PCR-DGGE fingerprinting, and microbial composition was obtained by cloning and sequencing of 16S rRNA genes. All assays were done in triplicate.

### Isolation and characterization of the hydrogen scavenger in IE culture.

A new enrichment series was set up by incubating IE culture with H_2_/CO_2_ (80%:20%, vol/vol, at 1.7 × 10^5^ Pa) plus 20 mM sulfonates (approximately 84% of BrES and 16% of isethionate) (Fig. S5 and Table S1). Isethionate has no known effect on methanogenic activity. It is a sulfonate that appears in the composition of some cosmetics and detergents and that can therefore end up in wastewater treatment plants. A total of 24 transfers and 3 sequential serial dilutions were performed in order to reduce microbial diversity and isolate hydrogen-consuming microorganisms. Composition of the stable enrichment culture, designated Desulf-BrES, was determined by direct sequencing of amplified partial 16S rRNA genes (sequences of 360 bp, obtained by Illumina sequencing), and confirmed by sequencing the nearly complete 16S rRNA genes (approximately 1,400 bp), obtained by cloning and Sanger sequencing. The purity of this culture was confirmed by microscopic observation of only one cell morphotype and by Illumina 16S rRNA sequencing, from which all sequences retrieved were assigned to Desulfovibrio sp. (Fig. S1).

Microbial growth was determined by measuring the optical density (wavelength at 600 nm). Hydrogen, VFA, and sulfide concentrations were monitored during the incubations. Control assays without H_2_/CO_2_ were performed. Additional experiments were performed with increasing concentrations of sulfonates (ranging from 5 to 50 mM), maintaining as constant the concentration of hydrogen/carbon dioxide, to investigate the relationship between sulfonate concentration and the amount of acetate produced. Growth in phosphate-buffered medium containing no bicarbonate nor carbon dioxide, with sulfonates as the sole carbon and energy sources, was also tested. Incubations with oleate, with and without sulfonates, were performed to investigate the ability of the hydrogen scavengers to degrade oleate. All incubations were done in triplicate assays.

Incubations of culture Desulf-BrES with sulfonates were performed under the following conditions: (i) with 20 mM BrES sterilized by autoclaving (composed of BrES plus isethionate) and with hydrogen (55 mM) as additional electron donor; (ii) with 4 mM sodium isethionate (98%; Sigma-Aldrich, St. Louis, MO) and with hydrogen as an additional electron donor; (iii) with 4 mM isethionate as the sole electron donor and electron acceptor; and (iv) with 20 mM BrES sterilized by filtering (aseptically using a syringe filter, 0.22-μm pore size) and with hydrogen as additional electron donor (Fig. S5). The optical density (600 nm) of the cultures and the concentrations of VFA, hydrogen, sulfide, bromide, and isethionate were followed over time.

### Analytical methods.

Hydrogen was measured by gas chromatography by using a MolSieve column (MS 13X, 80/100 mesh) connected to a thermal conductivity detector Bruker Scion 456 chromatograph (Bruker, Billerica, MA). Argon (30 ml · min^−1^) was the carrier gas, and injector, detector, and column temperatures were set at 100, 130, and 35°C, respectively. Methane was analyzed with a gas chromatograph (GC) (Chrompack 9000) equipped with a flame ionization detector (FID) and a 2 m × 1/8 in. Chromosorb 101 (80 to 120 mesh) column. Nitrogen was the carrier gas (30 ml · min^−1^), and column, injector, and detector temperatures were set at 35, 110, and 220°C, respectively.

Long-chain fatty acids (LCFAs) were first extracted with dichloromethane and esterified with 2-propanol prior to separation and quantification by gas chromatograph-flame ionization detector (GC-FID), as described by Neves et al. (40).

Liquid samples were centrifuged and filtered (0.22 μm) prior to VFA, bromide, isethionate, and sulfate determination. VFA concentrations were determined by high-performance liquid chromatography (HPLC; Jasco, Tokyo, Japan) using a Chrompack column (6.5 × 30 mm^2^) at 60°C and sulfuric acid (0.005 mol · liter^−1^) as mobile phase at a flow rate of 0.9 ml · min^−1^. VFA detection was done using a UV detector at 210 nm. Sulfide measurements were obtained using standard kits (Hach Lange, Düsseldorf, Germany). Bromide ion concentration was determined by the phenol red colorimetric method described in the *Standard Methods for the Examination of Water and Wastewater* (41). Isethionate and sulfate were analyzed by ion chromatography using a Dionex equipment (model DX-100) with a conductivity detector and an IonPac AS11-HC 4 × 250-mm column (Dionex, Sunnyvale, CA). Sodium hydroxide (10 mM) was used as the mobile phase at a flow rate of 1.4 ml · min^−1^, and the analysis was conducted at 20°C.

### Molecular methods.

Samples collected from oleate enrichment cultures were centrifuged and immediately frozen at −20°C in RNAlater (Sigma-Aldrich, St. Louis, MO). RNA was extracted by using the commercial kit FastRNA Pro Soil-Direct kit (MP Biomedicals, Solon, OH) and following the manufacturer’s instructions. cDNA was synthetized from RNA by SuperScript III reverse transcriptase (Thermo Fisher Scientific, Waltham, MA) using random primers.

Bacterial and archaeal 16S rRNA genes were amplified by PCR, using a *Taq* DNA polymerase (recombinant) (Invitrogen, Carlsbad, CA), for subsequent denaturing gradient gel electrophoresis (DGGE) analysis and cloning. Primer sets U968-f/1401-r (42) for Bacteria and A109(T)-f/515-r (43, 44) for Archaea were used for rRNA gene amplification prior to DGGE and cloning. For DGGE, a 40-bp GC clamp was added at the 5ʹ-end sequence of primers U968-f and 515-r (45). Reaction mixtures and PCR programs are described elsewhere (46). Size and yield of PCR products were estimated using the GeneRuler 1 kb Plus DNA ladder (Life Technologies, UK) via gel electrophoresis using agarose gels (1% wt/vol) stained with GreenSafe Premium (NZYTech, Lisbon, Portugal).

DGGE analysis of the PCR products was performed with the DCode system (Bio-Rad, Hercules, CA). Gels containing 8% (wt/vol) polyacrylamide (37.5:1 acrylamide:bis-acrylamide) were used with a linear denaturing gradient (30 to 60% for separation of bacterial amplicons and 30% to 50% for archaeal amplicons), with 100% of denaturant, corresponding to 7 M urea and 40% (vol/vol) formamide. Electrophoresis was performed for 16 h at 85 V and 60°C in a 0.5× Tris-acetate-EDTA buffer, and DGGE gels were stained with silver nitrate (47). PCR products previously purified with a NucleoSpin Extract II kit (Clontech Laboratories) were cloned into Escherichia coli JM109 (NZYTech, Lisbon, Portugal) cells by using the Promega pGEM-T Easy vector system (Promega, Madison, WI), as previously described (46). Clones with the correct size insert were further amplified for DGGE comparison with original sample profiles. Plasmids of transformants, corresponding to predominant bands in the DGGE community fingerprint, were purified with a Nucleo Spin extract II kit (Macherey-Nagel, Düren, Germany) and subjected to Sanger sequencing at Macrogen (Amsterdam, The Netherlands). DNA sequences were compared with those in the NCBI database by local alignment using nucleotide BLAST (https://blast.ncbi.nlm.nih.gov), and phylogenetic assignment was confirmed with the Ribosomal Database Project (RDP) classifier (48). Nucleotide sequences have been submitted to the European Nucleotide Archive under accession numbers LT992923 to LT992943, which are associated with the BioProject study accession number PRJEB25834.

Samples from culture Desulf-BrES were centrifuged and frozen at −20°C in phosphate-buffered saline (PBS) buffer. Total DNA was isolated with the commercial FastDNA SPIN kit for soil (MP Biomedicals, Solon, OH) and submitted to cloning and sequencing, following the procedure described for oleate enrichment cultures but using the primer set Bac27f/Uni1492r (44) to amplify 16S rRNA genes. Nucleotide sequences have been submitted to the European Nucleotide Archive under accession numbers LT991962 to LT991974, associated with the BioProject study accession number PRJEB25655.

Sequencing of 16S rRNA genes by Illumina MiSeq was performed to check the purity of the culture. Clone libraries, sequencing and data analysis were performed at the Research and Testing Laboratory (RTL; Lubbock, TX). The primer set 28F/388R (49, 50) was used in the amplification step. Detailed description of the procedure can be found in the supplemental material (supplemental methods). FASTQ files were submitted to the European Nucleotide Archive under the study accession number ERX2452693 (BioProject accession number PRJEB25655).

### Data availability.

The Desulfovibrio sp. strain (Desulf-BrES) was deposited in Leibniz Institute DSMZ-German Collection of Microorganisms and Cell Cultures under accession number DSM 108261. Nucleotide sequences described in this study have been submitted to the European Nucleotide Archive under accession numbers LT992923 to LT992943 (BioProject study accession number PRJEB25834) and accession numbers LT991962 to LT991974 (BioProject study accession number PRJEB25655). FASTQ files were submitted to the European Nucleotide Archive under the study accession number ERX2452693 (BioProject accession number PRJEB25655).

## Supplementary Material

Supplemental file 1
