# Supplementary material for: Inhibition Studies with 2-Bromoethanesulfonate Reveal a Novel Syntrophic Relationship in Anaerobic Oleate Degradation
Source: Appl Environ Microbiol. 2019 Jan 9;85(2):e01733-18. doi: 10.1128/AEM.01733-18 (PMC6328780; doi:10.1128/AEM.01733-18)
Supplement: Supplemental file 1 [file AEM.01733-18-s0001.pdf]

SUPPLEMENTARY MATERIAL

Supplementary Figures

Figure S1

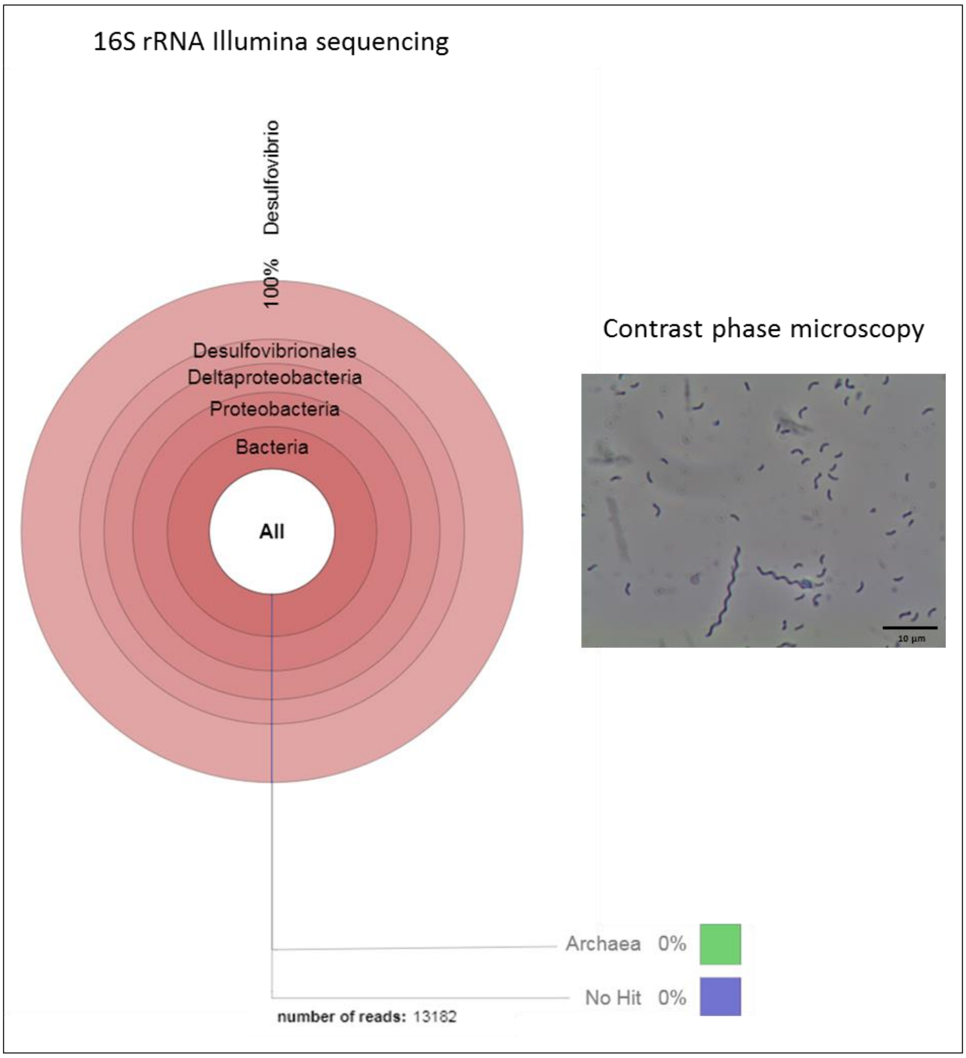

Fig. S1. Identification of *Desulfovibrio* sp. as the only microorganism growing on H_2/CO_2 and sulfonates, by total community 16S rRNA gene Illumina MiSeq sequencing. Phase contrast microscopy photographs showing *Desulfovibrio*-like cells growing either individually or forming long chains.

Figure S2

Identity (%)	clone 2	clone 3	clone 5	clone 11	clone 12	clone 24	clone 43	clone 44	clone 45	clone 46	clone 64	clone 69	clone 70	*Desulfovibrio aminophilus*
clone 2	100.00	99.32	99.19	99.26	99.26	99.39	99.32	99.12	99.46	99.32	99.39	99.12	99.26	98.24
clone 3	99.32	100.00	99.59	99.66	99.66	99.80	99.73	99.53	99.86	99.73	99.80	99.53	99.66	98.65
clone 5	99.19	99.59	100.00	99.59	99.53	99.66	99.59	99.39	99.73	99.73	99.80	99.39	99.53	98.51
clone 11	99.26	99.66	99.59	100.00	99.59	99.73	99.66	99.46	99.80	99.66	99.73	99.46	99.59	98.58
clone 12	99.26	99.66	99.53	99.59	100.00	99.73	99.66	99.46	99.80	99.66	99.73	99.46	99.59	98.58
clone 24	99.39	99.80	99.66	99.73	99.73	100.00	99.80	99.59	99.93	99.80	99.86	99.59	99.73	98.71
clone 43	99.32	99.73	99.59	99.66	99.66	99.80	100.00	99.53	99.87	99.73	99.80	99.53	99.66	98.65
clone 44	99.12	99.53	99.39	99.46	99.46	99.59	99.53	100.00	99.66	99.53	99.59	99.32	99.46	98.44
clone 45	99.46	99.86	99.73	99.80	99.80	99.93	99.87	99.66	100.00	99.86	99.93	99.66	99.80	98.78
clone 46	99.32	99.73	99.73	99.66	99.66	99.80	99.73	99.53	99.86	100.00	99.93	99.53	99.66	98.65
clone 64	99.39	99.80	99.80	99.73	99.73	99.86	99.80	99.59	99.93	99.93	100.00	99.59	99.73	98.71
clone 69	99.12	99.53	99.39	99.46	99.46	99.59	99.53	99.32	99.66	99.53	99.59	100.00	99.46	98.44
clone 70	99.26	99.66	99.53	99.59	99.59	99.73	99.66	99.46	99.80	99.66	99.73	99.46	100.00	98.58
Desulfovibrio aminophilus	98.24	98.65	98.51	98.58	98.58	98.71	98.65	98.44	98.78	98.65	98.71	98.44	98.58	100.00

Number of mismatches	clone 2	clone 3	clone 5	clone 11	clone 12	clone 24	clone 43	clone 44	clone 45	clone 46	clone 64	clone 69	clone 70	*Desulfovibrio aminophilus*
clone 2	0	9	11	10	10	8	8	12	7	9	8	12	10	22
clone 3	9	0	6	5	5	3	3	7	2	4	3	7	5	17
clone 5	11	6	0	6	7	5	5	9	4	4	3	9	7	19
clone 11	10	5	6	0	6	4	4	8	3	5	4	8	6	18
clone 12	10	5	7	6	0	4	4	8	3	5	4	8	6	18
clone 24	8	3	5	4	4	0	2	6	1	3	2	6	4	16
clone 43	8	3	5	4	4	2	0	6	1	3	2	6	4	16
clone 44	12	7	9	8	8	6	6	0	5	7	6	10	8	20
clone 45	7	2	4	3	3	1	1	5	0	2	1	5	3	15
clone 46	9	4	4	5	5	3	3	7	2	0	1	7	5	17
clone 64	8	3	3	4	4	2	2	6	1	1	0	6	4	16
clone 69	12	7	9	8	8	6	6	10	5	7	6	0	8	20
clone 70	10	5	7	6	6	4	4	8	3	5	4	8	0	18
Desulfovibrio aminophilus	22	17	19	18	18	16	16	20	15	17	16	20	18	0

Number of gap opens	clone 2	clone 3	clone 5	clone 11	clone 12	clone 24	clone 43	clone 44	clone 45	clone 46	clone 64	clone 69	clone 70	*Desulfovibrio aminophilus*
clone 2	0	1	1	1	1	1	2	1	1	1	1	1	1	4
clone 3	1	0	0	0	0	0	1	0	0	0	0	0	0	3
clone 5	1	0	0	0	0	0	1	0	0	0	0	0	0	3
clone 11	1	0	0	0	0	0	1	0	0	0	0	0	0	3
clone 12	1	0	0	0	0	0	1	0	0	0	0	0	0	3
clone 24	1	0	0	0	0	0	1	0	0	0	0	0	0	3
clone 43	2	1	1	1	1	1	0	1	1	1	1	1	1	4
clone 44	1	0	0	0	0	0	1	0	0	0	0	0	0	3
clone 45	1	0	0	0	0	0	1	0	0	0	0	0	0	3
clone 46	1	0	0	0	0	0	1	0	0	0	0	0	0	3
clone 64	1	0	0	0	0	0	1	0	0	0	0	0	0	3
clone 69	1	0	0	0	0	0	1	0	0	0	0	0	0	3
clone 70	1	0	0	0	0	0	1	0	0	0	0	0	0	3
Desulfovibrio aminophilus	4	3	3	3	3	3	4	3	3	3	3	3	3	0

Figure S2. Comparison of the thirteen 16S rRNA gene sequences obtained from culture Desulf-BrES (designated by clone 2, 3, 5, 11, 12, 24, 43, 44, 45, 46, 64, 69 and 70) in terms of identity (%), number of mismatches and number of gap opens, determined by pairwise sequence alignment. The 16S rRNA sequence from *Desulfovibrio aminophilus* strain ALA-3 (NR_024916.1), the closest relative to culture Desulf-BrES, is included for comparative proposes.

Figure S3

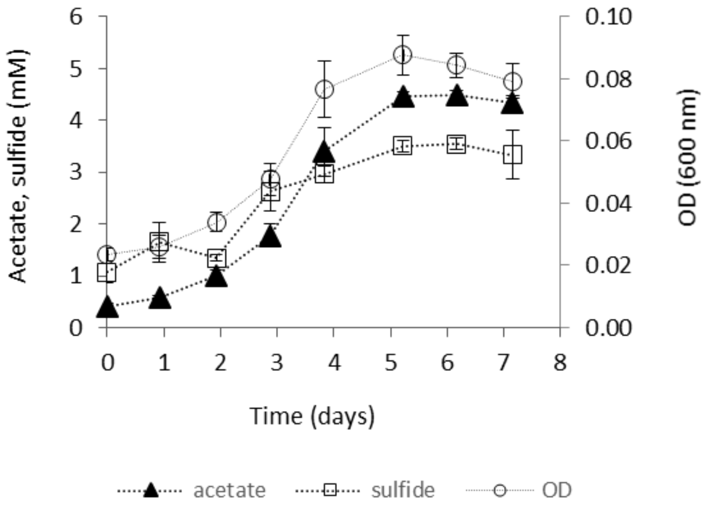

Fig. S3. Products of sulfonates (BrES + isethionate) conversion by culture Desulf-BrES in phosphate buffered medium. Growth is represented by the increase of the optical density measured at 600 nm. The results presented are the average and standard deviations for triplicate assays.

Figure S4

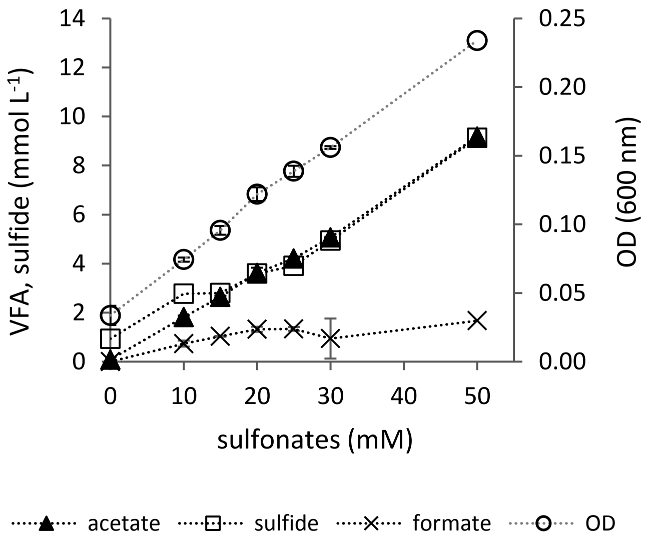

Fig. S4. Effect of increasing the concentrations of sulfonates (BrES plus isethionate) in microbial growth and in the production of acetate, formate and sulfide. All the parameters were determined at the end of the incubations. The results presented are the average and standard deviations for triplicate assays.

Figure S5

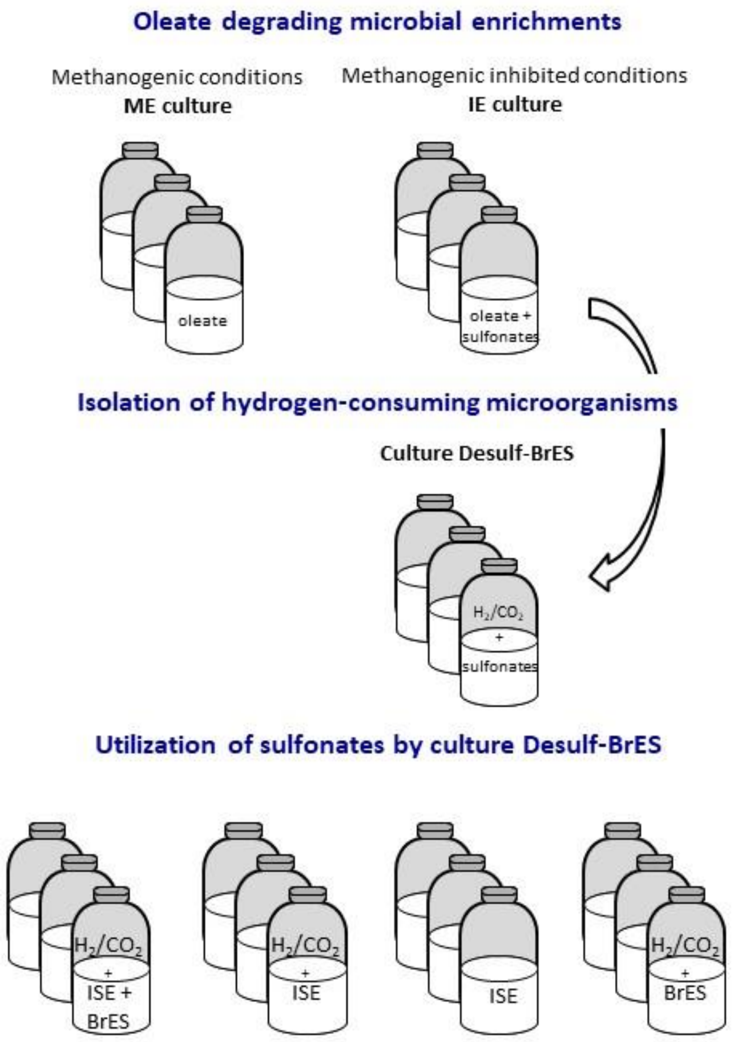

Fig. S5. Schematic representation of the enrichment cultures obtained in this study. ISE: isethionate; BrES: 2-bromoethanesulfonate.

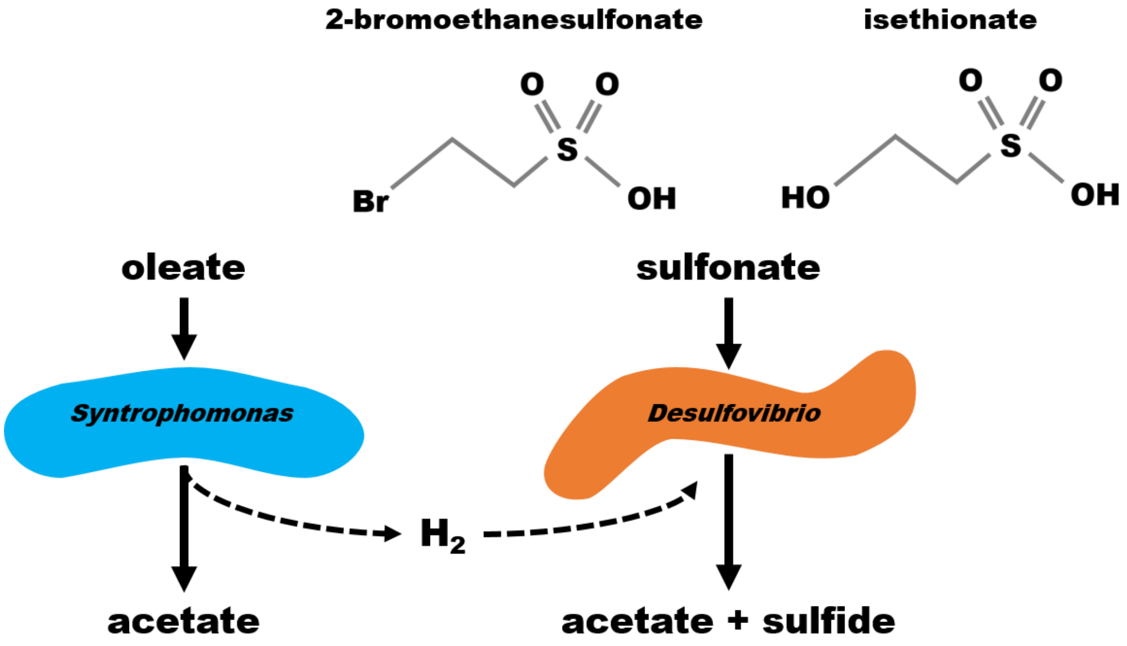

Fig. S6. Schematic representation of the syntrophic conversion of oleate with sulfonates

Supplementary Tables

Table S1

Table S1. Release of bromide from BrES after one and three sterilization cycles, which reflects the extent of BrES hydrolysis after autoclavation. The results presented are the average and standard deviations for triplicate assays.

	Bromide released from BrES (%)
BrES solution autoclaved once	16 ± 1.5
BrES solution autoclaved three times	52 ± 3.8

Supplementary Methods

Sequencing of 16S rRNA amplicons by Illumina MiSeq

DNA amplification was done in a two-step process using a forward primer constructed with (5'-3') the Illumina i5 sequencing primer (TCGTCGGCAGCGTCAGATGTGTATAAGAGACAG) and the 28F (GAGTTTGATCNTGGCTCAG) primer (1), and a reverse primer constructed with (5'-3') the Illumina i7 sequencing primer (GTCTCGTGGGCTCGGAGATGTGTATAAGAGACAG) and the 388R (TGCTGCCTCCCGTAGGAGT) primer (2). Amplifications were performed in 25 ul reactions with Qiagen HotStar Taq master mix (Qiagen Inc, Valencia, California), 1ul of each 5uM primer, and 1ul of template on a ABI Veriti thermocycler (Applied Biosytems, Carlsbad, California) under the following thermal profile: 95°C for 5 min, then 25 cycles of 94°C for 30 sec, 54°C for 40 sec, 72°C for 1 min, followed by one cycle of 72°C for 10 min and 4°C hold. Products from the first stage amplification were added to a second PCR based on qualitatively determine concentrations. Primers for the second PCR were designed based on the Illumina Nextera PCR primers as follows: Forward - AATGATACGGCGACCACCGAGATCTACAC[i5index]TCGTCGGCAGCGTC and Reverse - CAAGCAGAAGACGGCATACGAGAT[i7index]GTCTCGTGGGCTCGG. The second stage amplification was run the same as the first stage except for 10 cycles. Amplification products were visualized with eGels (Life Technologies, Grand Island, New York), pooled equimolar and each pool was size selected in two rounds using Agencourt AMPure XP (BeckmanCoulter, Indianapolis, Indiana) in a 0.7 ratio for both rounds. Size selected pools were quantified using the Quibit 2.0 fluorometer (Life Technologies) and loaded on an Illumina MiSeq (Illumina, Inc. San Diego, California) 2x300 flow cell at 10pM. Sequences obtained underwent denoising and chimera checking before microbial

diversity analysis. The forward and reverse reads were merged together using the PEAR Illumina paired-end read merger (3). Prefix dereplication was performed with USEARCH algorithm (4). Sequences with less than 100 bp in length were discarded; clustering was performed at a 4% divergence using USEARCH cluster algorithm (4) and clusters with less than 2 members removed from the data set. OUTs were selected with UPARSE OUT selection algorithm (5). Chimera checking was performed using the UCHIME chimera detection software (6) executed in *de novo* mode and all chimeric sequences were removed. Microbial diversity analysis and taxonomic assignment was done by running sequences through the USEARCH global alignment program and using a database of high quality sequences derived from NCBI that is maintained in Research and Testing Laboratory (http://www.medicalbiofilm.org).

References

1. **Handl S**, **Dowd SE**, **Garcia-Mazcorro JF**, **Steiner JM**, **Suchodolski JS**. 2011. Massive parallel 16S rRNA gene pyrosequencing reveals highly diverse fecal bacterial and fungal communities in healthy dogs and cats. FEMS Microbiol Ecol **76**:301–310.

2. **MacIntyre DA**, **Chandiramani M**, **Lee YS**, **Kindinger L**, **Smith A**, **Angelopoulos N**, **Lehne B**, **Arulkumaran S**, **Brown R**, **Teoh TG**, **Holmes E**, **Nicoholson JK**, **Marchesi JR**, **Bennett PR**. 2015. The vaginal microbiome during pregnancy and the postpartum period in a European population. Sci Rep **5**:8988.

3. **Zhang J, Kobert K, Flouri T, Stamatakis A**. 2014. PEAR: a fast and accurate Illumina Paired-End reAd mergeR. Bioinformatics **30**:614–620.

4. **Edgar RC**. 2010. Search and clustering orders of magnitude faster than BLAST. Bioinformatics **26**:2460–2461.

5. **Edgar RC**. 2013. UPARSE: highly accurate OTU sequences from microbial amplicon reads. Nat Methods **10**:996–998.

6. **Edgar RC, Haas BJ, Clemente JC, Quince C, Knight R**. 2011. UCHIME improves sensitivity and speed of chimera detection. Bioinformatics **27**:2194–2200.
